# Lobectomy vs. MRgLITT in Temporal Lobe Epilepsy (TLE): A Pilot Study Investigating Vestibulo-Spatial Functions

**DOI:** 10.3390/jcm14010156

**Published:** 2024-12-30

**Authors:** Milos Dordevic, Kiana Assady Looyeh, Friedhelm C. Schmitt, Notger G. Müller

**Affiliations:** 1 Degenerative and Chronic Diseases of the Faculty of Health Sciences (FGW), University Potsdam, 14469 Potsdam, Germany; 2 Department of Neurology, Otto-von-Guericke-University Magdeburg, 39120 Magdeburg, Germany

**Keywords:** TLE, MRgLITT, balance, spatial, vestibular

## Abstract

**Background**: About 65 million people worldwide are affected by epilepsy, with temporal lobe epilepsy being the most common type resistant to drugs and often requiring surgical treatment. Although open surgical approaches, such as temporal lobectomy, have been the method of choice for decades, minimally invasive MRgLITT has demonstrated promising results. However, it remains unknown whether patients who underwent one of these two approaches would show better performance on vestibulo-spatial tasks. **Methods**: Twenty-seven patients were included in three different groups: (1) MRgLITT (37.0 ± 15.1 years, two females), (2) R-OP (44 ± 15.7 years, five females) and (3) No-OP (43 ± 11.2 years, three females)—with no significant differences in age, disease duration and number of medications. Groups were compared on their performance in three vestibular-dependent tests: (1) clinical balance test (CBT), (2) triangle completion test (TCT) and (3) rotational memory (RM) test. **Results**: Significantly better performance of MRgLITT patients, in comparison to the other two groups (R-OP and No-OP), was found for the TCT. The other tests revealed no significant differences between the groups. **Conclusions**: Patients who underwent MRgLITT performed significantly better on the vestibular-dependent spatial orientation task (TCT) compared to those who underwent temporal lobectomy (R-OP) and non-operated patients. Speculations about reasons for such an effect—including minimal invasiveness with less “collateral damage”, influence of operated side, timing of surgery, sample heterogeneity and others—need to be assessed in detail in larger-scale, prospective longitudinal studies.

## 1. Introduction

About 50 million people suffer from epilepsy worldwide [1], with temporal lobe epilepsy being the most common drug-resistant type [2]. Its prevalence amounts to about 10.4/10,000 people per year, with up to 71 percent of these being drug-resistant and therefore considered for surgery [3].

For drug-resistant TLE, since the 1950s, the anterior temporal lobectomy (ATL) has been the gold standard surgical method [4,5]; however, all open surgical procedures have several inherent risks, including infections and hemorrhages [6]. Since just over a decade ago, a stereotactic laser ablation—also known as MRI-guided laser interstitial therapy (MRgLITT)—has also been applied to ablate neural tissue in TLE cases [7]. Minimal invasiveness, preservation of white matter tracts, similar rates of seizure freedom to that of ATL and reduced overall costs are some aspects recommending its application [5,8].

To date, very few studies have compared the functional and cognitive benefits following the two surgical procedures—ATL and MRgLITT. Better naming and recognition functions have been previously reported in patients who underwent MRgLITT [9]. Additionally, non-verbal memory is better preserved, including performance on spatial tests, such as the Rey Complex Figure Test, for patients undergoing MRLITT [10].

In this respect, several inputs from the temporal lobe are critical for successful spatial navigation, such as from the hippocampal place cells and Papez circuit head direction cells, as well as the grid cells located in the entorhinal cortex; moreover, these regions receive inputs from the vestibular system [11,12]. In addition, several studies have found a link between the vestibular system and temporal lobe regions, where the inputs from the former are processed [13,14,15]. Furthermore, results of our previous study revealed significant impairments in vestibulo-MTL functions in patients suffering from TLE, including in terms of path integration and rotational memory [16]. In accordance with those results, studies evaluating path integration abilities—assessed using the triangle completion test (TCT)—reported worse performance in patients who underwent a temporal lobectomy [17,18]. However, no previous study has compared the outcomes of ATL vs. MRgLITT with regard to vestibular-dependent spatial functions—namely path integration, rotational memory and balancing.

Therefore, the aim of this study was to compare post-surgery performance on three vestibular-dependent tests (the triangle completion test, rotational memory test and clinical balancing test) between three groups of patients who underwent the following procedures: (I) ATL, (II) MRgLITT and (III) no surgery. Based on previous studies, we hypothesized significantly better performance of patients who underwent MRgLITT compared to the other two groups of this post-surgery assessment.

## 2. Materials and Methods

### 2.1. Participants and Study Design

This study was organized as cross-sectional with one factor: group (MRgLITT, R-OP and No-OP). All measurements in this study took place from 2018 until 2022 in the DZNE (German Center for Neurodegenerative Diseases) in Magdeburg. In total, twenty-seven patients (Table 1) were selected for the study, with nine in each group—there were no significant differences in age between patients in the MRgLITT (37.0 ± 15.1 years, two females), R-OP (44 ± 15.7 years, five females) and No-OP (43 ± 11.2 years, three females) groups. Patients in the MRgLITT group were prospectively collected and assessed (within one month following surgery) for this study, whereas participants of the other two groups were retrospectively included and analyzed (see [16]). Eligible patients for this study were all those with a TLE-diagnosis aged from 18 to 80 years old. All TLE patients were recruited through referral from the Epilepsy Department of the Otto von Guericke University Clinic for Neurology in Magdeburg. The epileptogenic zone [19] was determined using video electroencephalography (EEG) monitoring (i.e., seizure semiology and ictal EEG) and neuroimaging evaluation (MRI and positron emission tomography (PET)), which included the detection of epileptogenic lesions such as hippocampal sclerosis. No patient had clinical signs of a cerebellar syndrome, typically seen in patients with drug-induced vestibular dysfunction. Patients suffered either from short-term (several minutes) focal aware or non-aware seizures.

### 2.2. Surgical Procedure

Both surgical procedures—standard resection and MRgLITT—were carried out at the Neurosurgery Department of the Otto von Guericke University Clinic in Magdeburg. For details on the MRgLITT surgical procedure, see [20]. In brief, in 2018, the CE certification for this treatment option was issued in Germany. Open microsurgical resection and stereotactic laser thermal ablation were discussed in detail with the selected patients. Due to the lower invasiveness and the continued possibility of being able to resort to a respective procedure (step-by-step procedure), the patients opted for this intervention. The ablation of the mesial part of the amygdala-hippocampal complex was carried out in three individual steps. Preoperative planning of the ablation volume was carried out using a stereotactic planning procedure that included high-resolution MRI, functional MRI and positron emission tomography and simulated brachytherapy. The exact position and size of the epileptogenic focus or target volume for ablation was determined using the imaging data. In addition, the optimal access route and the exact placement of the laser fiber were calculated. This minimized the risk of damaging neighboring healthy tissue. With the help of preoperative planning, a fiber-optic laser probe with a diameter of about one millimeter was inserted and fixed in the brain via a small hole in the skull, usually under general anesthesia. Intraoperatively, the position of the laser probe was checked using real-time MRI (with a delay of approx. 3–4 s) to enable continuous adjustment of the energy output and the position of the laser probe. An MRI was performed postoperatively to compare the actual ablation volume with that in the preoperative planning.

### 2.3. Tests

#### 2.3.1. Clinical Balance Test (CBT)

For more details on the clinical balance test (CBT), please refer to our previously published studies [14,16,21]. In brief, patients were assessed on several standing, including stable and unstable surfaces, and walking conditions; further sub-conditions included assessments with open and closed eyes. Overall, CBTs consisted of 30 assessment items, with the maximal number of points reaching 90—unsatisfactory performance on each of 30 items received 0, whereas optimal performance received 3 points.

#### 2.3.2. Triangle Completion Test (TCT)

Similarly, further details on the triangle completion test (TCT) can be found in our previously published studies [14,16,21]. This test assessed participants’ non-visual spatial orientation abilities. Briefly, the room floor was marked with six triangular paths—with three oriented towards the left and three towards the right. Therefore, these paths produced in total three pairs of triangular paths, each consisting of 60°, 90°, and 120° turning angles. Participants were first walked (active) and then pushed (passive, wheelchair) along each of the abovementioned paths—this resulted in 12 trials per participant in total (three to the left and three to the right multiplied by the two conditions—walking and pushed in wheelchair). The final objective in each of the conditions for participants was to walk back to the starting point using the shortest possible (direct) way. The outcomes/variables of interest were the distance and the angular error.

#### 2.3.3. Rotational Memory (RM)

The rotational memory test was also described in detail elsewhere [14,21]. Participants were first seated in a rotatory chair (Interacoustics, Middelfart, Denmark). The chair was programmed to rotate left and right (about the vertical axis in the transverse plane). For the whole duration of the test, participants were blindfolded and their ears were covered. In each condition (consisting of one or more rotations), participants were asked to rotate back to the initial position—this was achieved by telling the examiner first the direction and afterwards the extent of rotation (the examiner rotated the chair manually according to participants’ instructions, until their “initial position” had been reached). The whole test consisted of eight conditions in total: twice one rotation, twice two, twice four and twice eight rotations. The outcome of interest was angular distance between the original initial position and the initial position selected by participants on each of the conditions. After each trial, the chair was automatically rotated back to the original initial position.

### 2.4. Statistical Analysis

Analysis of all behavioral data was performed using SPSS v.21 (IBM, Armonk, NY, USA). All data were checked for assumptions of normality and homogeneity of variance. To inspect the within-group pre-post differences, a dependent *t*-test or Wilcoxon test (when assumptions were not fulfilled) was applied. In the tables, the respective means with standard deviations are presented. A Chi-square test was performed to analyze the frequency of left- and right-sided TLE between groups. In addition, the effect sizes (partial Eta squared and Cohen’s d) are listed. In the figures, the respective means with a 95% confidence interval of the difference between means are depicted. The data were collected by author K.A-L., who was blinded to data analysis, and analyzed by author M.D., who was blinded to the data collection.

## 3. Results

The datasets of twenty-seven patients were analyzed, with nine in each of the three groups (MRgLITT, R-OP and No-OP). No significant group differences could be found with regards to age, disease duration and number of medications. Two patients from the MRgLITT group could not be tested on RM at the post-test due to technical issues, leaving seven complete datasets from this test for the analysis. Nine complete datasets were obtained from the temporal resection group of patients (R-OP) and the group which did not receive surgery (No-OP).

**Figure 1:**
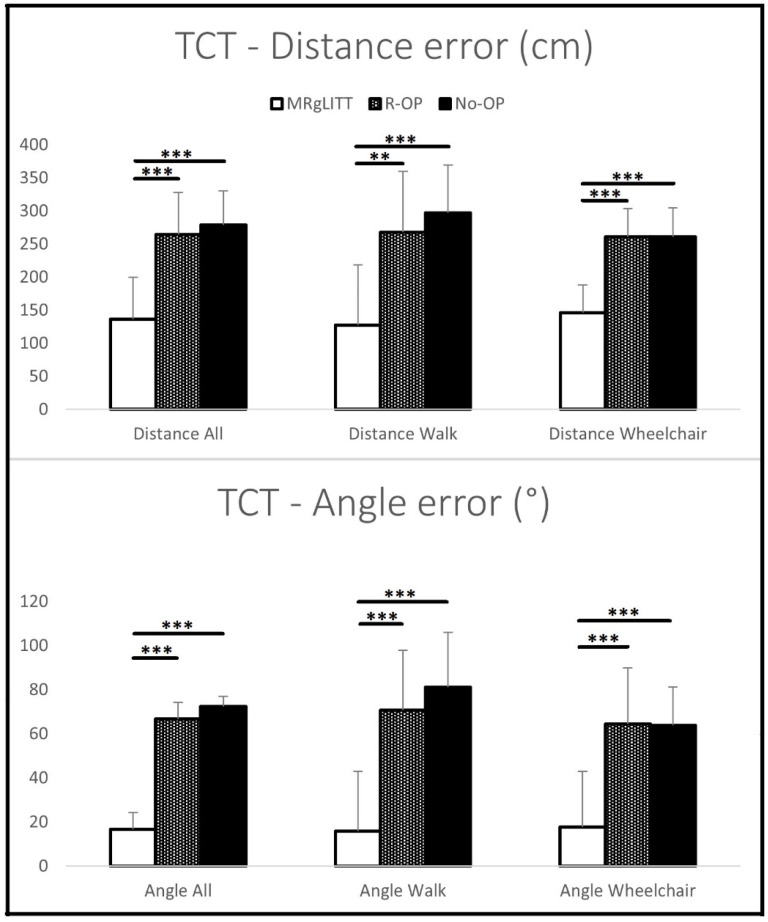
Errors on the TCT for both conditions (Walk and Wheelchair) for three groups of patients—(I) MRgLITT-operated, (II) operated with temporal resection and (III) not operated patients; ** *p* < 0.01, *** *p* < 0.001; TCT—triangle completion test. Error bars represent 95% confidence intervals.

As summarized in Table 2, patients in the MRgLITT group performed significantly better on the TCT compared to patients in the R-OP and No-OP groups (Figure 1). Therefore, they demonstrated better ability to return to the point of origin of the triangular path. In other words, they had smaller errors in both distance (point of origin to the point where participant ended up) and angle (walking direction towards the point of origin).

No significant difference in performance between groups could be found for RM and CBT. However, despite non-significant differences on these tests, the analysis revealed medium to large effect sizes on all conditions (Table 3), which might point to the hindering effect of the small sample of patients in each group. Further sub-group analyses were not performed because of the relatively small sample size.

## 4. Discussion

This study compared vestibulo-spatial abilities in three groups of TLE patients—(I) received MRgLITT, (II) received ATL-resection (R-OP) and non-operated (No-OP). Their performance on three tests was recorded—namely, the clinical balancing test (CBT), triangle completion test (TCT) and rotational memory (RM) test. As hypothesized, patients who had undergone the less invasive MRgLITT procedure demonstrated better performance on the TCT compared to the other two groups of patients. This effect cannot be attributed to other group differences such as age, as this difference was not significant. However, no significant differences on the other two tests could be detected. Nevertheless, relatively large effect sizes speak in favor of MRgLITT on these tests, too.

TLE is the most common drug-resistant type of epilepsy [2]. Studies on structural changes have reported both cortical atrophy and shifts towards brain network regularization in TLE [1,2]; moreover, adaptive memory reorganization to the healthy hemisphere has been reported [22]. Animal studies revealed that brain lesions, and not the epileptic condition per se, might cause hippocampal-dependent spatial memory deficits [23]. In addition, spatial memory in animals is altered already during the seizure-free latent period, which further correlated with a decrease in the power of theta oscillations [24]. Furthermore, epileptic mice fail to efficiently develop a spatial search strategy [25]. Poor spatial navigation demonstrated by TLE patients on the human version of the Morris Water Maze task is significantly associated with longer disease duration, younger age at epilepsy onset and lower intelligence level [26]. Our earlier study revealed significantly inferior performance of TLE patients compared to healthy controls in all three of the behavioral tests used in the current study, with large effect sizes [16]. Also, postural function, measured using the Sway Index (SI), may worsen in adults suffering from generalized epilepsy compared to controls [27]. Indeed, one study suggested the ictal involvement of cortical-subcortical structures known to be part of the vestibular integration network, such as temporo-parieto-occipital regions—these regions are well known for processing vestibular information, including those in the ipsilateral cerebellar hemisphere [28].

In general, epilepsy surgery remains the method of choice for drug-resistant TLE patients, since in this way about half of the patients remain completely seizure free [20]. Although ATL is considered the gold standard, minimally invasive MRgLITT has been considered a safe and effective treatment for TLE patients since its introduction just over a decade ago [29]. Other advantages of MRgLITT include a decreased hospitalization time, length of procedure time and analgesic requirement when compared to open surgery, together with similar seizure-free rates [30,31]. However, a recent review summarized and reported various rates of decline and improvements in functional outcomes following MRgLITT; this might point towards the need for more standardized methods for accurate capturing and quantifying risks associated with MRgLITT [8,32].

Earlier studies on TLE patients who underwent (mainly right-sided) ATL reported a decrease in consistency of path integration and a systematic under-registration of linear displacement, also assessed via TCT—this suggested that the right temporal lobe plays a role in idiothetic spatial memory [17,18]. To date, no such data were available for MRgLITT, making the current study the first one to compare the two surgical approaches with respect to path integration abilities. The reasons for better performance on the TCT by MRgLITT patients compared to R-OP and No-OP can only be speculated upon based on the findings of previous studies. A recent study has shown that the integrity of some MTL structures—which are important predictors of memory decline—such as the uncinate fasciculus, are only severed during resection and not during laser surgery [33]. Patients undergoing ATL experience a greater reduction in visuospatial memory metrics, including performance on the Rey Complex Figure Test, compared to patients undergoing stereotactic laser surgery [10]. Some functions, including naming and recognition tasks, are known to be spared in TLE patients undergoing laser surgery; these deficits are considered to occur as “collateral damage” during resection surgery [9], which might have happened in our study as well. Nevertheless, this could explain better performance of the patients in the MRgLITT group compared to the OP group but not to those in the non-OP group. Numerous studies on both animals and humans have already described the critical role of the right medial temporal lobe in processing spatial inputs [34,35,36]—therefore, it can be speculated that better performance was observed due to MRgLITT being performed predominantly on right-sided TLE patients. However, for such a statement to be confirmed or refuted, future studies on larger and more homogeneous samples are necessary.

Despite the important findings reported in this study, it also contains several limitations. It has been suggested that analysis in larger cohorts with extra-long-term follow-up is needed for identification of good prognostic factors and other postsurgical outcomes [37]—this study was neither longitudinal nor did it contain a large cohort of patients in each group; therefore, the results must be interpreted with caution. Nevertheless, it was meaningful to run a plausibility study on a small number of participants before organizing a larger scale study, which requires significantly more resources. In addition, patients’ visual abilities were not tested in this study—visual field deficits are more common in patients treated with open temporal lobectomy compared to laser-based minimally invasive procedures [4,38]. Furthermore, in our study, patients in the MRgLITT group were all tested within one week following surgery, which was not the case with the ATL group, who performed the tests from several months to several years following surgery. In addition, all groups were relatively inhomogeneous with respect to gender, operated side, presence of sclerosis before surgery, etc. Finally, the lack of randomization in this study impairs its strength, allowing for numerous other factors to be considered when interpreting the results. Therefore, in order to draw more relevant conclusions, future research should include larger cohorts, adopt longitudinal designs and implement randomized controlled trials (RCTs), while controlling for confounding factors such as visual deficits, gender distribution and lateralization.

## 5. Conclusions

In conclusion, this study provides evidence of varying functional outcomes from three TLE-patient groups: (I) MRgLITT operated (MRgLITT), (II) ATL operated (R-OP) and (III) non-operated (No-OP). As hypothesized, the patients who had undergone MRgLITT performed significantly better on the path integration task (TCT) compared to the R-OP group, perhaps due to minimal invasiveness and less “collateral damage” or to the group predominantly consisting of right-sided TLE patients. Although on the other two tasks this difference did not reach significance, relatively large effect sizes spoke in favor of MRgLITT as well. However, considering the cross-sectional nature and relatively small sample size in this study, together with the lack of a randomization procedure, the results must be interpreted with caution. Future longitudinal RCT studies on larger and more homogeneous cohorts are required for drawing more confident conclusions.

## Figures and Tables

**Table 1:** Characteristics of patients; AEDs—Anti-epileptic drugs.

Patient (P)	Surgery (OP)	Age (y)	Gender	Disease Duration (y)	Lateralisation	No. of AEDs
P1	MRgLITT	22	M	15	Right	2
P2	MRgLITT	64	F	44	Left	3
P3	MRgLITT	57	F	21	Bilateral	5
P4	MRgLITT	39	M	20	Right	1
P5	MRgLITT	35	M	29	Right	3
P6	MRgLITT	37	M	29	Right	4
P7	MRgLITT	30	M	3	Right	3
P8	MRgLITT	17	M	24	Right	3
P9	MRgLITT	32	M	2	Left	3
P1	R-OP	64	M	45	Left	4
P2	R-OP	58	F	23	Right	2
P3	R-OP	27	M	12	Left	2
P4	R-OP	47	F	38	Left	2
P5	R-OP	29	M	22	Left	3
P6	R-OP	38	M	10	Left	1
P7	R-OP	53	F	50	Left	1
P8	R-OP	23	F	15	Left	2
P9	R-OP	61	F	55	Right	3
P1	No-OP	38	M	35	Left	3
P2	No-OP	34	M	8	Left	3
P3	No-OP	58	F	6	Bilateral	2
P4	No-OP	61	M	24	Bilateral	1
P5	No-OP	32	F	25	Left	2
P6	No-OP	45	F	6	Left	1
P7	No-OP	46	M	17	Left	3
P8	No-OP	48	M	24	Right	1
P9	No-OP	29	M	6	Right	2

**Table 2:** Results for all TCT conditions from MRgLITT vs. R-OP vs. No-OP group comparisons; SD—standard deviation, **—*p* < 0.01, ***—*p* < 0.001, ^§^—d > 0.8 or η^2^ > 0.14.

Test	Condition(s)		Mean ± SD MRgLITT	Mean ± SD R-OP	Mean ± SD No-OP	Post-hoc MRgLITT vs. R-OP *p*-Value Effect Size (d)	Post-hoc MRgLITT vs. No-OP *p*-Value Effect Size (d)
Triangle Completion Test (TCT)	Angle	All Conditions	16.8 ± 5.6	66.9 ± 23.3	72.4 ± 17.7	<0.001 *** 2.947 ^§^	<0.001 *** 4.230 ^§^
		Walk	15.8 ± 8.9	70.7 ± 30.5	81.1 ± 31.2	<0.001 *** 2.434 ^§^	<0.001 *** 2.856 ^§^
		Wheelchair	17.7 ± 3.5	64.5 ± 26.5	64.0 ± 16.8	<0.001 *** 2.478 ^§^	<0.001 *** 2.604 ^§^
	Distance	All Conditions	136.7 ± 56.2	264.6 ± 56.2	279.0 ± 46.0	0.001 *** 2.078 ^§^	<0.001 *** 2.776 ^§^
		Walk	127.2 ± 69.6	268.0 ± 96.7	297.3 ± 74.1	0.008 ** 1.669 ^§^	<0.001 *** 2.368 ^§^
		Wheelchair	146.1 ± 45.7	261.2 ± 58.9	260.7 ± 42.4	<0.001 *** 2.185 ^§^	<0.001 *** 2.604 ^§^

**Table 3:** Results for all CBT and RM conditions from MRgLITT vs. R-OP vs. No-OP group comparisons; SD—standard deviation, ^§^—d > 0.8 or η^2^ > 0.14.

Test	Condition(s)	Mean ± SD MRgLITT	Mean ± SD R-OP	Mean ± SD No-OP	ANOVA *p*-Value	Effect Size (η^2^)
Rotational Memory (RM)	All Conditions	31.9 ± 17.2	51.5 ± 24.3	47.2 ± 15.1	0.142	0.159 ^§^
	One Rotation	16.4 ± 7.2	42.4 ± 24.7	38.3 ± 23.5	0.055	0.245 ^§^
	Two Rotations	47.9 ± 34.1	49.2 ± 33.5	56.9 ± 45.4	0.871	0.012
	Four Rotations	32.1 ± 24.7	45.6 ± 29.5	48.1 ± 20.2	0.429	0.074
	Eight Rotations	31.4 ± 31.1	63.1 ± 55.6	45.6 ± 41.9	0.386	0.083
Clinical Balance Test (CBT)	All Conditions	64.3 ±15.1	56.7 ± 8.3	54.2 ± 16.3	0.281	0.100
	Open Eyes	52.7 ± 13.3	46.6 ± 7.8	44.4 ± 12.9	0.314	0.092
	Closed Eyes	11.7 ± 2.3	10.1 ± 1.9	9.8 ± 3.7	0.318	0.091

## Data Availability

Data are available under special permission.
